# Untargeted metabolomics and physicochemical analyses reveal metabolic mechanisms underlying quality modification in sour porridge fermented with *Lacticaseibacillus paracasei* subsp. *paracasei* GXUN74722

**DOI:** 10.3389/fmicb.2026.1868992

**Published:** 2026-07-01

**Authors:** Guofang Liu, Wen Huang, Shenghua Luo, Yajian Qin, Shanshan Wei, Yuying Huang, Weixin Huang, Yuting Qu, Yuanyuan Man, Huizhao Su

**Affiliations:** 1 Guangxi Key Laboratory for Polysaccharide Materials and Modifications, School of Marine Sciences and Biotechnology, Guangxi Minzu University, Nanning, Guangxi, China; 2 The First Affiliated Hospital of Guangxi Medical University, Nanning, Guangxi, China; 3 Guangxi Key Laboratory of Immunology and Metabolism for Liver Diseases, Nanning, Guangxi, China

**Keywords:** inoculated fermentation, *Lacticaseibacillus paracasei* subsp. paracasei, natural fermentation, sour porridge, untargeted metabolomics

## Abstract

Traditional fermented sour porridge is a characteristic traditional fermented food in Guangxi Zhuang Autonomous Region, China, featuring distinctive sensory flavor and abundant microbial diversity. In this study, a strain of *Lacticaseibacillus paracasei* subsp. *paracasei* GXUN74722 was isolated and identified from traditional fermented sour porridge collected from Fusui County, Chongzuo City, Guangxi Zhuang Autonomous Region, China. The strain exhibited high fermentation efficiency in sour porridge production. Using pH value and titratable acidity as core evaluation indices, the fermentation conditions for GXUN74722-inoculated sour porridge were systematically optimized. The optimal fermentation parameters were determined as follows: fermentation time of 72 h, inoculation dose of 12.5%, fermentation temperature of 37 °C, and glucose addition (2% glucose solution) at a volume fraction of 15%. Inoculated fermented sour porridge with strain GXUN74722 induced significant differences in the physicochemical characteristics and metabolic profiles of sour porridge relative to naturally fermented sour porridge. Untargeted metabolomics was applied to compare low-molecular-weight metabolite variations between naturally fermented and inoculated fermented sour porridge samples. Metabolomic analysis confirmed obvious discrepancies in metabolite composition between the two groups. Organic acids and their derivatives were the most predominant metabolites, accounting for 34.83% of the total identified metabolites, followed by organoheterocyclic compounds (17.53%), and lipids and lipid-like molecules (16.54%). A total of 1,312 metabolites were annotated in all samples, including 83 identified differential metabolites. These differential metabolites were primarily classified into amino acids, peptides and analogues, carbohydrates and carbohydrate conjugates, lipids and lipid-like molecules, as well as benzene and substituted derivatives. KEGG pathway enrichment analysis showed that five metabolic pathways were significantly altered, including ABC transporters, galactose metabolism, pentose phosphate pathway, carbohydrate digestion and absorption, and glycerophospholipid metabolism. Overall, inoculation with *L. paracasei* subsp. *paracasei* GXUN74722 reshaped the synthesis and accumulation profiles of metabolites in sour porridge. The observed physicochemical and metabolic shifts may underpin enhanced fermentation stability as well as modified sensory and nutritional attributes of fermented sour porridge. Since the present work only characterized physicochemical and metabolomic variations without dedicated sensory evaluation or *in vivo* functional tests, further validations are required to verify actual quality improvement and prospective health-promoting potentials of inoculated fermented sour porridge. These findings provide fundamental theoretical basis and technical references for the industrial development and standardized production of fermented sour porridge.

## 1 Introduction

As a traditional fermented cereal food, sour porridge is commonly consumed in Guangxi Zhuang Autonomous Region, Shanxi Province, Inner Mongolia Autonomous Region, and Shaanxi Province of China. Primarily manufactured from rice, millet, maize, and broomcorn millet through spontaneous microbial fermentation, this food boasts favorable nutritional profiles and reputed digestive-promoting functions (Ma et al., 2024; Ignat et al., 2020). Traditional fermented sour porridge harbors rich lactic acid bacteria diversity dominated by genera *Lactobacillus*, *Acetobacterium* and *Weissella*, whose metabolic activities shape the characteristic flavor and textural attributes of the final product (Liu et al., 2010; Jaiswal et al., 2022). Nevertheless, natural fermentation remains the predominant production mode at present and suffers from inherent drawbacks including monotonous flavor, prolonged fermentation cycles, and inconsistent product quality. Directed inoculation fermentation technology can effectively strategy to mitigate these limitations (Guo H. et al., 2020; Wang J. et al., 2021; Castellone et al., 2021). The application of selected high-performance lactic acid bacteria strains can endow fermented cereals with distinctive flavors and optimized nutritional composition while improving the microbial safety of final fermented foods.

Lactic acid bacteria (LAB) are a food-grade microorganism with generally recognized as safe status, exhibiting probiotic properties such as facilitating nutrient absorption, modulating intestinal microbiota homeostasis, reducing serum cholesterol, and modulating host immune status (Dahiya and Nigam, 2022; Xiong et al., 2023; Wang et al., 2022). LAB synthesize and degrade diverse nutritional substrates during metabolic processes, which remodels food nutritional composition and improves the bioavailability of nutrients for human digestion (Srikham et al., 2021; Peredo-Lovillo et al., 2020). Most LAB-driven non-alcoholic fermented cereal foods feature acidic sensory characteristics. As the main raw material for sour porridge fermentation, rice is rich in carbohydrates, starch, protein, free amino acids and other nutrients, and supplies energy and nutritional support for more than half of the global population (Duan et al., 2024; Zhang et al., 2014). Previous studies have demonstrated that microbial fermentation endows rice sensory properties, enriches nutrient composition, and improves nutrient digestibility (Chen et al., 2023). Multiple LAB exhibit prominent proteolytic activity and hydrolyze macromolecular proteins into readily absorbable small molecules (Usal et al., 2024). Following LAB fermentation, cereal matrices display modified edible quality and elevated antioxidant capacity, accompanied by increased total acid yield, decreased reducing sugar and accumulation of numerous volatile aroma compounds (Xiao et al., 2026; Li et al., 2024). Wang et al. (2024a, 2024b) isolated *Levilactobacillus brevis* M-10 from naturally fermented sour porridge and verified its *in vitro* cholesterol-lowering potential as well as its fermentative and digestive physiological features. These findings provide theoretical support for developing novel sour porridge with modified functional characteristics. Different LAB exert significant regulatory effects on the flavor characteristics of fermented rice products by modulating volatile and non-volatile metabolite profiles. They also improve the bioavailability of intrinsic vitamins and minerals in cereals, thereby further enhancing their nutritional value (Lee et al., 2019; Carrizo et al., 2020). Moreover, LAB produce organic acids during fermentation to lower medium pH and suppress the proliferation of most spoilage and pathogenic microbes (Gao et al., 2019). Furthermore, LAB-synthesized bacteriocins and functional metabolites can effectively extend food shelf life and strengthen food safety (Ibrahim et al., 2021).

In recent years, untargeted metabolomics has emerged as an indispensable analytical tool to decipher metabolic shifts during food fermentation, enabling comprehensive profiling of metabolite categories and abundance variations. Liu et al. (2021) integrated transcriptomic and proteomic analyses to explore the molecular mechanisms underlying flavor formation and antioxidant properties of fermented rice porridge. Their results revealed that the up-regulated genes and proteins of *Lactobacillus paracasei* H4-11 were significantly enriched in multiple KEGG pathways, including starch and sucrose metabolism, pyruvate metabolism, amino acid and nucleotide glucose metabolism, glycolysis, and fructose metabolism. Ghamry et al. (2023) reported that fermentation with *Lactobacillus apis* significantly enhanced the antioxidant activity of wheat bran and modulated the contents of free amino acids and organic acids. Xu et al. (2023) observed remarkable changes in nutrients, flavor, and antioxidant activities of brown rice after LAB fermentation, where starch and protein degradation generated polysaccharides, bioactive peptides and free amino acids. Zhang Y. et al. (2021) used a mixed starter composed of *Lactobacillus paracasei* L1 and *Lactobacillus casei* YQ336 to ferment corn and its by-products, and detected several bioactive substances, including 1-hydroxy-2-naphthalene carboxylic acid (a naphthalene phenolic compound with anti-inflammatory activity), isoprene (with antibacterial and hypotensive effects), and epicatechin (with anti-tumor properties). However, few studies have focused on revealing the differences between naturally fermented and inoculated fermented sour porridge based on untargeted metabolomics.

In this study, liquid chromatography-mass spectrometry (LC–MS)-based untargeted metabolomics was employed to compare metabolite profiles and relevant metabolic pathways between naturally fermented sour porridge and sour porridge inoculated with *L. paracasei* subsp. *paracasei* GXUN74722. Metabolite annotation and pathway enrichment were performed relying on public databases including KEGG and HMDB. The resulting metabolomic data were analyzed to offer empirical guidance for subsequent optimization of sour porridge fermentation technology. The findings may serve as a preliminary theoretical foundations for further quality evaluation and targeted metabolic regulation of sour porridge products.

## 2 Materials and methods

### 2.1 Strains and medium

*Lacticaseibacillus paracasei* subsp. *paracasei* GXUN74722 (*L. paracasei* subsp. *paracasei* GXUN74722) was isolated from traditional fermented sour porridge, which was collected from five random farm households in Fusui County, Chongzuo City, Guangxi Zhuang Autonomous Region, China (107°3ˊE, 22°11ˊN). *L. paracasei* subsp. *paracasei* GXUN74722 was cultured in MRS medium (Qingdao Haibo Biotechnology Co., Ltd., China).

### 2.2 Isolation and identification of strains

Appropriate amount of sour porridge samples were serially diluted to form a concentration gradient, and the diluents were spread on CaCO_3_-MRS solid medium, followed by incubation at 37 °C for 48 h. Single colonies with distinct calcium-dissolving circles and consistent morphological features were selected, and purified via repeated subculturing to acquire pure cultures. Based on the acid-producing capability as the screening criterion, the pH value of the fermentation broth (cultured in MRS broth medium for 24 h) was measured; and isolates with fermentation broth pH below 4.0 were selected as the target LAB in this experiment.

Strain GXUN74722 was taxonomically identified based on 16S rRNA gene sequence analysis. Genomic DNA of strain GXUN74722 was extracted, and the 16S rRNA gene was amplified by PCR with universal primers 27F (5’-AGAGTTTGATCMTGGCTCAG-3′) and 1541R (5’-AAGGAGGTGATCCAGCC-3′). After detection by 1% agarose gel electrophoresis, the PCR products were sequenced by Shanghai Sangon Biotech (Shanghai, China). The sequencing data obtained were further analyzed for homology alignment on EzBioCoud website^1^ and the phylogenetic tree was subsequently constructed using MEGA 11 software.

Cell morphological characteristics of the strain were observed with a SUPRA 55 Sapphire scanning electron microscope (SEM, Carl Zeiss, Germany). Gram-positive and Gram-negative bacteria were identified using a Gram staining kit (Guangdong Huankai Microbial Technology Co., Ltd., Guangzhou, China), following the manufacturer’s protocols, consisting of crystal violet staining, alcohol decolorization and sand yellow counterstaining. Stained bacterial cells were observed under a light microscope to determine the Gram-staining results.

### 2.3 Optimization of strain culture conditions

Determination of bacterial growth curves (Shao et al., 2023): The seed culture of strain GXUN74722 was inoculated into MRS broth medium at an inoculation dose of 2% (v/v). The culture was incubated aerobically at 37 °C with continuous shaking at 200 r/min for 24 h. Bacterial culture samples were collected at 2 h intervals to determine the absorbance value (OD_600_) and pH value of the fermentation broth. The growth curve and dynamic pH variation curve of the strain were generated based on the acquired experimental data.

Effect of temperature on strain growth (Ye et al., 2020): The seed culture of strain GXUN74722 was inoculated into MRS broth medium at an inoculation dose of 2% (v/v), and cultured at 200 r/min for 24 h under a series of temperature conditions (16, 20, 25, 30, 37, 40, and 45 °C). After incubation, the pH value and absorbance value (OD_600_) of the fermentation cultures were determined. All temperature treatments were performed in three parallel replicates to ensure the reliability of the experimental data.

Effect of glucose concentration on strain growth (Zhang Z. et al., 2021): The seed culture of strain GXUN74722 was inoculated into MRS broth medium supplemented with different glucose concentrations (0, 10, 20, 30, 40, and 50%) at an inoculation dose of 2% (v/v). The cultures were incubated at 37 °C with constant shaking at 200 r/min for 24 h. Subsequently, the pH value and absorbance value (OD_600_) of the fermentation broth were determined. Each treatment was performed in three parallel replicates to ensure the reliability of the experimental data.

### 2.4 Optimization of fermentation conditions for sour porridge

The rice with plump grains, free of empty shell and impurities was selected and rinsed with distilled water repeatedly until no suspended solids were observed in the eluate. After draining the water completely, a certain amount of the treated rice was taken at a water-to-rice ratio of 5:1, subjected to sterilization at 105 °C for 5 min, and then cooled to room temperature for subsequent use. The control group was fermented in open containers to allow natural colonization of ambient microbes instead of artificial strain inoculation, serving as blank control for strain fermentation comparison; this system differs from conventional traditional spontaneous fermentation with unsterilized rice. An appropriate volume of strain GXUN74722 bacterial suspension was inoculated into the prepared rice medium, sealed, and incubated in a constant temperature incubator for fermentation to obtain the final sour porridge product. Using pH value and titratable acidity as the core evaluation indices, single-factor experiments were performed to optimize key fermentation parameters, including fermentation time, inoculation dose, glucose (2% glucose solution) addition volume fraction in rice medium, and fermentation temperature, respectively.

Optimization of fermentation time: The strain GXUN74722 (OD_600_ = 1.0) was inoculated into the rice medium with an inoculation dose of 12.5% and glucose (2% glucose solution) addition volume fraction of 12.5%, and fermented at 37 °C for 0, 24, 48, 72, 96 and 120 h, respectively. After fermentation, the pH value and titratable acidity of sour porridge samples were determined to screen out the optimal fermentation time.

Optimization of inoculation dose: The strain GXUN74722 (OD_600_ = 1.0) was inoculated into the rice medium with glucose (2% glucose solution) addition volume fraction of 12.5% at gradient inoculation doses of 2.5, 5, 10, 12.5 and 15%, and fermented at 37 °C for 72 h. The pH value and titratable acidity of the fermented sour porridge were determined to confirm the optimal inoculation dose for sour porridge fermentation.

Optimization of glucose concentration in rice medium: The strain GXUN74722 was inoculated at a fixed inoculation dose of 12.5% into rice medium with gradient glucose (2% glucose solution) addition volume fractions (2.5, 5, 10, 12.5 and 15%), and fermented at 37 °C for 72 h. The pH and titratable acidity of the samples were subsequently measured for the optimization of glucose concentration for fermentation.

Optimization of fermentation temperature: The strain GXUN74722 was inoculated into the rice medium with an inoculation dose of 12.5% and optimal glucose concentration, the inoculated rice medium was fermented at gradient temperatures of 28, 31, 34, 37, and 40 °C for 72 h, respectively. The pH value and titratable acidity of sour porridge from each temperature group were determined to confirm the optimal fermentation temperature.

### 2.5 Analysis of physicochemical properties of sour porridge

Strain GXUN74722 was inoculated into the rice medium for sour porridge fermentation according to the aforementioned fermentation protocol at 37 °C for 72 h, with naturally fermented sour porridge assigned as the control group. Samples of both naturally fermented and inoculated fermented sour porridge were collected at 0, 24, 48 and 72 h post-inoculation, respectively.

pH value measurement: pH value of sour porridge samples was determined in accordance with the Chinese national standard GB 5009.237-2016 using a calibrated pH meter, and all measurements were conducted in biological triplicate with results documented accordingly.

Titratable acidity determination: Titratable acidity of sour porridge samples was determined according to the Chinese national standard GB 5009.239-2016. The samples were evenly stirred to ensure homogeneity. A 10.0 g aliquot of the homogenized samples was accurately weighed, transferred into a beaker, and mixed with 100 mL of double-distilled water. Subsequently, 2–4 drops of phenolphthalein indicator solution (10 g/L) were added to the mixture. The resulting solution was titrated with 0.1 mol/L NaOH standard solution until a faint reddish color appeared and persisted for 30 s without fading; the volume of consumed sodium hydroxide (NaOH) was recorded precisely. The titratable acidity of the samples was calculated according to Equation 1.


$$X=\frac{c\times (V-V_{0})\times 10}{m\times 0.1}$$ (1)

Where: *X*: titratable acidity of the sample, °T; c: molar concentration of NaOH standard solution, mol/L; V: volume of NaOH consumed in the titration, mL; V_0_: volume of NaOH consumed in the blank titration, mL; 10: 10 g of sample; m: actual mass of the specimen, g; 0.1: standard molar concentration of NaOH defined by the acidity theory, mol/L.

Lactic acid content determination: Lactic acid content was measured using a commercially available assay kit (Nanjing Jiancheng, China), following the manufacturerʼs instructions. All assays were carried out in triplicate.

### 2.6 Metabolomics analysis of naturally fermented and inoculated fermented sour porridge based on LC–MS

Sample preparation: Sour porridge was fermented under optimized conditions: 12.5% inoculation dose, 15% glucose supplementation, and incubation at 37 °C for 72 h. Two experimental groups were established in this study, including naturally fermented sour porridge as the control group (CK), and inoculated fermented sour porridge as the experimental group (EG). Six independent biological replicates were prepared for each group via parallel fermentations under identical processing parameters. Upon fermentation termination, 5.0 g of each sour porridge sample was accurately weighed and transferred into sterile centrifuge tubes. All prepared samples were sent to Shanghai Winnerbio Technology Co., Ltd. (Shanghai, China; www.innerbio.cn) for untargeted metabolomics detection using ultra-high-performance liquid chromatography-quadrupole-Orbitrap mass spectrometry (UPLC-Q-Extractive Orbitrap MS).

Metabolite extraction: Frozen sour porridge samples were thawed at 4 °C, and 25 mg aliquot of each homogenized samples was accurately weighed and transferred into a 1.5 mL centrifuge tube. Next, 800 μL prechilled extraction solution (−20 °C; methanol:acetonitrile: Water = 2:2:1, v:v:v) and 10 μL mixed internal standards (containing d_3_-Leucine, ^13^C_9_-Phenylalanine, d_5_-Tryptophan and ^13^C_3_-Progesterone) were added to the centrifuge tube, followed by two small grinding beads. Samples were homogenized in a tissue grinder at 50 Hz for 5 min, subjected to ultrasonic extraction in a 4 °C water bath for 10 min, and incubated at −20 °C refrigerator for 1 h. After incubation, the mixture was centrifuged at 25000 rpm and 4 °C for 15 min. A total of 600 μL supernatant was carefully collected and transferred to a freeze vacuum concentrator for drying. The dried residues were reconstituted in 600 μL reconstitution solvent (methanol:water = 1:9, v:v), vortex-mixed for 1 min, and ultrasonically processed at 4 °C water bath for 10 min. Following a second centrifugation at 25000 rpm and 4 °C for 15 min, the resulting supernatant was retained for subsequent metabolomic measurement.

UPLC-Q-Extractive Orbitrap MS analysis: Chromatographic separation was performed on a Vanquish ultra-high-performance liquid chromatography (UHPLC) system equipped with a HILIC column and a 4 °C refrigerated autosampler. The injection volume was set at 2 μL, column temperature maintained at 25 °C, and flow rate fixed at 0.3 mL/min. Mobile phase A consisted of aqueous solution supplemented with 25 mM ammonium acetate and 25 mM ammonia, while mobile phase B was pure acetonitrile. The linear gradient elution procedure was configured as follows: 0–1.5 min, 98% phase B maintained; 1.5–12 min, phase B linearly decreased from 98 to 2%; 12–14 min, 2% phase B maintained; 14–14.1 min, phase B linearly increased from 2 to 98%; 14.1–17 min, 98% phase B maintained for column requilibration. Quality control (QC) samples were prepared alongside test samples throughout LC–MS detection. Metabolite peak intensities of QC samples were applied to monitor LC–MS system stability, instrument performance, as well as assay repeatability of the entire detection system (Qing et al., 2025).

Electrospray ionization (ESI) was employed for the detection of each sour porridge in both positive and negative ion modes. The samples were separated by UHPLC and analyzed using a Q Exactive Series Mass Spectrometer (Thermo Fisher Scientific). The parameters of the ESI source and mass spectrometry were set as follows: atomized gas (Gas1): 60; auxiliary heating gas (Gas2): 60; curtain gas (CUR): 30 psi; ion source temperature: 600 °C; spray voltage (ISVF): ± 5,500 V (for positive and negative ion modes, respectively); primary mass-to-charge ratio (m/z) detection range: 80–1,200 Da; primary resolution: 60000; scan accumulation time: 100 ms. For the secondary mass spectrometry, a segmented acquisition method was adopted, with the following parameters: scanning range: 70–1,200 Da; secondary resolution: 30000; scan cumulative time: 50 ms; dynamic exclusion time: 4 s.

### 2.7 Data processing and statistical analyses

All experimental data were sorted and collated using Microsoft Office Excel 2021. Statistical analysis was performed by SPSS 21.0 software. One-way analysis of variance was employed for multi-group comparisons, and the least significant difference (LSD) test was used for variance comparison. Independent samples *t*-test was applied for pairwise comparisons between two groups. *p* < 0.05 indicated a significant difference. All graphs were generated using Origin 2022 software. All experiments were performed in triplicate, and the experimental results were presented as mean ± standard deviation (SD).

Untargeted metabolomics raw LC–MS data were preprocessed via metaX software to screen qualified metabolites and acquire valid quantitative data for subsequent metabolomic analysis. The identified metabolites were annotated against the Kyoto Encyclopedia of Genes and Genomes (KEGG) and Human Metabolome Database (HMDB), yielding annotation information including KEGG ID, HMDB ID, metabolite taxonomy, and participating metabolic pathways.

Metabolite identification confidence was classified according to Metabolomics Standards Initiative (MSI) classification system: Level 1 identification was defined by matching retention time, accurate mass and MS/MS fragmentation with authentic reference standards; Level 2 was assigned for metabolites with high-score MS/MS matching against HMDB databases with mass tolerance<5 ppm for precursor ions and <15 ppm for fragment ions; Level 3 was annotated based solely on accurate molecular mass without MS/MS spectrum; Level 4 represented unknown compounds with only elemental composition prediction.

## 3 Results and discussion

### 3.1 Screening and identification of lactic acid bacteria

**Figure 1:**
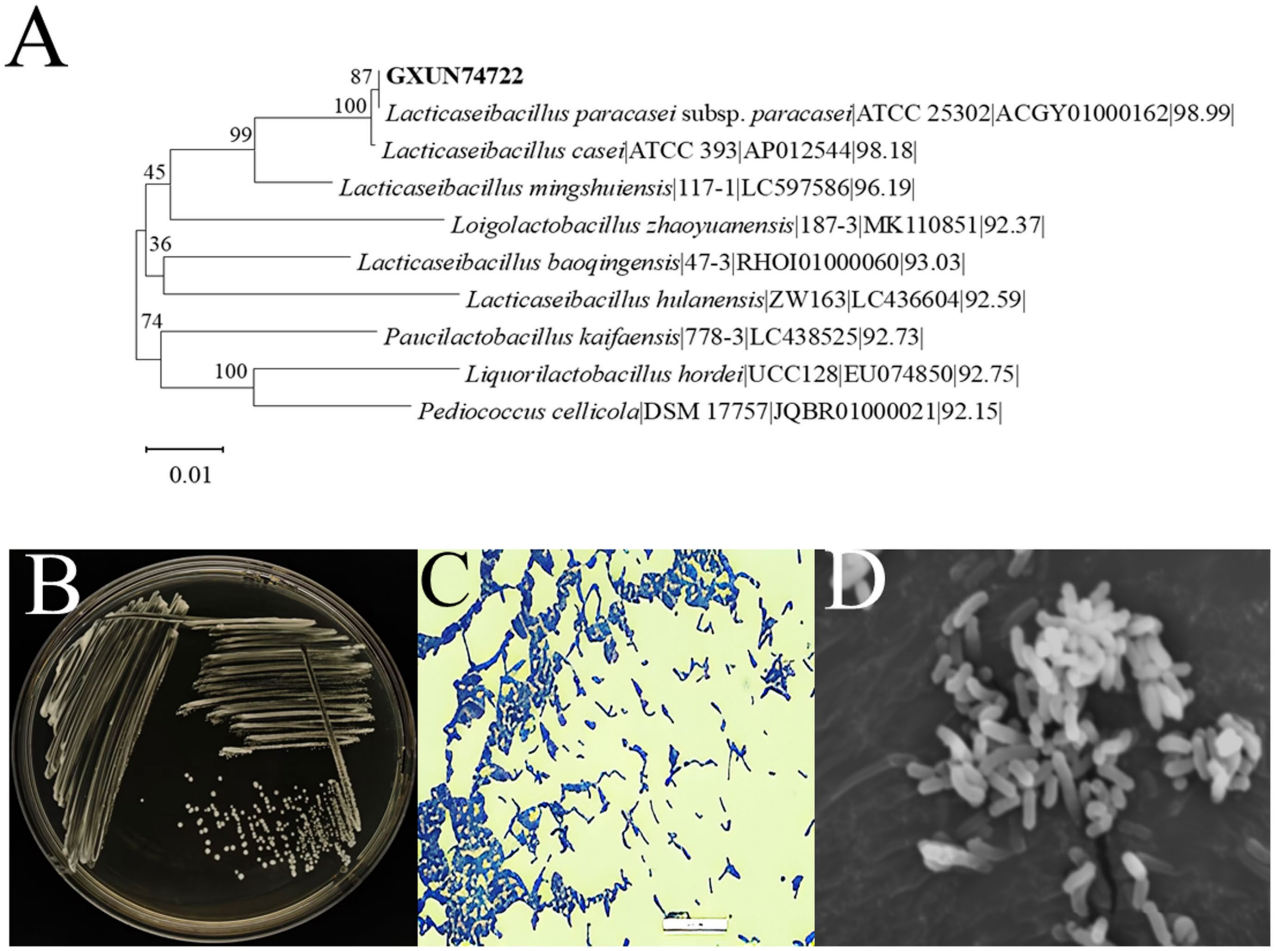
Phylogenetic tree and morphological characteristics of strain GXUN74722. **(A)** Neighbor-joining phylogenetic tree; **(B)** Colonial characteristics of strain GXUN74722 on MRS agar plates; **(C)** Gram staining of strain GXUN74722 observed under light microscopy (1000×); **(D)** Cellular ultrastructure of strain GXUN74722 observed under scanning electron microscopy (6500×).

In this study, a total of 11 acid-producing strains were isolated from five sour porridge samples collected from Fusui County, Chongzuo City, Guangxi Zhuang Autonomous Region, China, all of which exhibited negative catalase activity. These 11 isolated and identified strains were all LAB belonging to the genus *Lacticaseibacillus* within the phylum Firmicutes. Among them, strain 1C-6 was selected as the target strain for further investigation. After 24 h cultivation in MRS broth medium, the pH value of fermentation broth was determined to be 3.87 ± 0.02. A phylogenetic tree was constructed for this strain, and the results revealed that strain 1C-6 shared 98.99% sequence similarity with *Lacticaseibacillus paracasei* subsp. *paracasei* ATCC 25302, with the two strains clustered on the same phylogenetic branch (Figure 1A). Therefore, this strain was identified and designated as *Lacticaseibacillus paracasei* subsp. *paracasei* GXUN74722 (Peng et al., 2021; Hwanhlem et al., 2022), and had been deposited in the China General Microbiological Culture Collection Center (CGMCC No. 33135). When cultured on MRS agar plates, strain GXUN74722 formed medium-sized, round, milky-white colonies with neat, raised, and moist edges (Figure 1B). Gram staining confirmed that the strain GXUN74722 was Gram-positive (Figure 1C), and scanning electron microscopy (SEM) observation further demonstrated its typical rod-shaped cellular morphology (Figure 1D).

### 3.2 Optimization of culture conditions for strain GXUN74722

**Figure 2:**
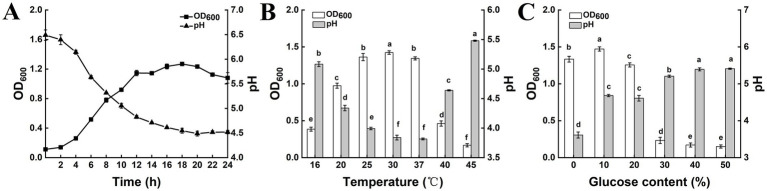
Growth and acid-producing characteristics of strain GXUN74722 under different culture conditions. **(A)** Growth curve and acid-producing performance of strain GXUN74722; **(B)** Effect of temperature on the growth and acid-producing of strain GXUN74722; **(C)** Effect of glucose content in MRS broth on the growth and acid-producing of strain GXUN74722.

Growth curve analysis is a fundamental approach for characterizing the growth kinetics of microbial strains and determining their optimal culture period (Huang et al., 2024). The acid-producing capability is a key indicator for evaluating the application potential of LAB as fermentation starter cultures. Lactic acid is the primary organic acid synthesized by various probiotics stains and has been widely applied in the food, chemical, medical and other industries. In the present study, the growth dynamics and acid-producing characteristics of strain GXUN74722 were investigated by monitoring the growth curve and fermentation broth pH at different incubation time points, aiming to systematically assess its fermentation performance and acid-producing capability. As shown in Figure 2A, strain GXUN74722 entered the exponential growth phase after 2 h of cultivation, with a rapid increase in cell proliferation. The active growth of the strain was accompanied by continuous acid accumulation and a sharp decline in fermentation broth pH value. The declining rate of pH gradually slowed after 18 h of cultivation, and the final pH stabilized at 4.52 at 24 h of fermentation. These findings indicate that strain GXUN74722 exhibits excellent acid-producing capability. The acidic microenvironment generated by LAB metabolism can effectively inhibit the proliferation of spoilage and pathogenic microorganisms during food production and storage, thereby extending the shelf life of fermented food products (Jung et al., 2021).

Temperature is a critical environmental factor governing the life activities of microorganisms. Temperatures deviating from the optimal growth range can affect the intracellular enzymes activity of LAB, thereby impairing cell growth, the yield and quality of microbial metabolites, and further affecting the acidity, flavor, and texture of fermented products (He X. N. et al., 2025). As shown in Figure 2B, strain GXUN74722 exhibited a broad growth temperature tolerance ranging from 16 °C to 45 °C, with the optimal growth and acid-producing performance observed at 30–37 °C. Within this optimal temperature range, strain GXUN74722 proliferated rapidly, achieving an OD_600_ value of 1.35–1.42 and the fermentation broth pH decreasing to 3.81–3.84. When the culture temperature was elevated to 40 °C, a significant reduction in OD_600_ value and a marked increase in pH were observed, indicating that the growth and metabolic activities of strain GXUN74722 were substantially inhibited under temperatures above 40 °C.

Carbohydrates are indispensable carbon sources and energy substrates for LAB fermentation, which support microbial growth and sustain normal fermentative metabolism (Chen et al., 2012). Appropriate sugar concentrations are critical for LAB proliferation and metabolism, while excessive or insufficient sugar supply can negatively affect fermentation efficiency. Specifically, excessively high sugar concentrations increase water molecule binding affinity. On the contrary, insufficient sugar induces nutritional limitation. This condition impedes substrate transmembrane transport, restricts the growth and proliferation of LAB, and diminishes their fermentative performance. Additionally, low sugar levels will compromise the sensory quality of the finished fermented products (Adams et al., 1987). As a readily metabolizable monosaccharide, glucose can be directly absorbed and catabolized by LAB to generate lactic acid. In this study, different glucose concentrations in MRS broth were set to explore the carbon source adaptability of strain GXUN74722 (Figure 2C). The growth rate and acid-producing capacity of strain GXUN74722 were significantly improved when the glucose concentration in MRS broth increased to 10%. Stable acid production was observed with glucose concentrations ranging from 10 to 20%, whereas a gradual decrease in bacterial growth was detected. When the glucose concentration exceeded 20%, strain GXUN74722 almost lost its acid-producing ability, and its growth was significantly suppressed. Collectively, these results suggest that the optimal glucose concentration for the growth, proliferation, and acid-producing metabolism of strain GXUN74722 is 0–10%.

### 3.3 Optimization of fermentation conditions for sour porridge

**Figure 3:**
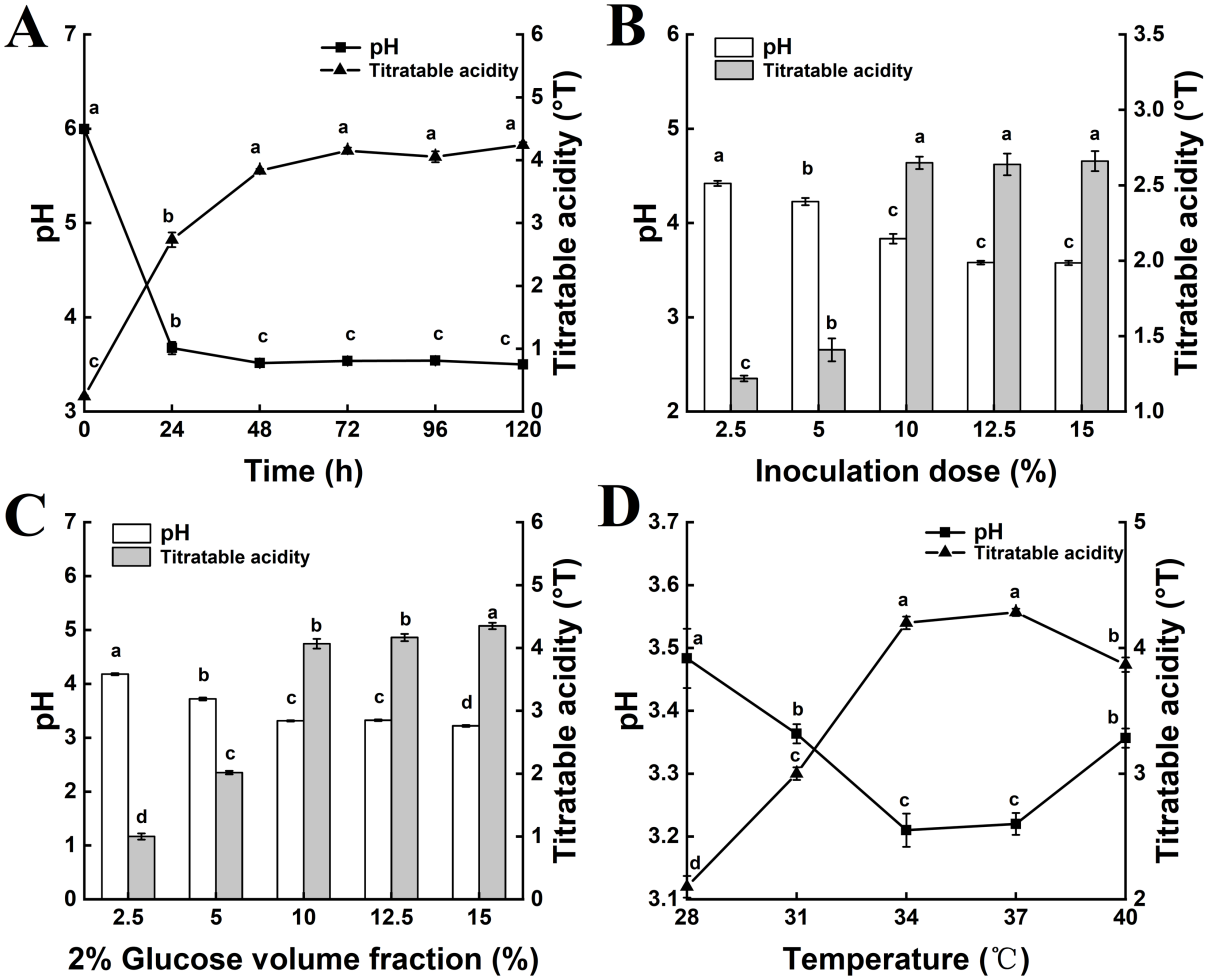
Optimization of fermentation parameters for sour porridge inoculated with strain GXUN74722. **(A)** Fermentation time; **(B)** Inoculation dose; **(C)** 2% glucose volume fraction; **(D)** Fermentation temperature (all on pH and titratable acidity).

Fermentation time is a critical determinant of production efficiency, manufacturing cost, and overall fermentation cycle, making the screening of probiotic strains with potent fermentation-shortening capacity a key research focus in fermented food biotechnology (Park et al., 2018). The acid-producing capacity of LAB serves as an important indicator for evaluating fermentation characteristics, directly reflecting the metabolic activity of microorganisms during fermentation (Gao et al., 2021). In this study, pH value and titratable acidity were selected as core physicochemical indicators to optimize the fermentation time of sour porridge inoculated with strain GXUN74722. As illustrated in Figure 3A, prolonged fermentation contributed to continuous LAB proliferation and progressive accumulation of organic acids, accompanied by a gradual reduction in pH value and a steady elevation in titratable acidity. After 72 h of fermentation, the pH value and titratable acidity of the sour porridge inoculated with strain GXUN74722 reached a relatively stable state, with a pH value of 3.54 ± 0.02 and titratable acidity of 4.15 ± 0.05 °T. This phenomenon is attributed to the hydrolysis of partial starch in cereal raw materials into reducing sugars, which are subsequently catabolized by LAB to produce organic acids. Meanwhile, the accumulated organic acids solubilize water-soluble proteins in the fermentation matrix, effectively increasing free amino acids content of fermentation broth (Olayinka et al., 2020). In conclusion, the optimal fermentation time for GXUN74722-inoculated sour porridge was determined to be 72–96 h. To balance time cost and production efficiency, 72 h was selected as the standardized fermentation time for subsequent experiments.

Inoculation dose significantly governs fermentation rate and the comprehensive quality of fermented products, and an optimal inoculation dose can both shorten the fermentation cycle and facilitate the accumulation of nutritional components in the fermented products (Guo G. et al., 2020). As shown in Figure 3B, incremental increase in inoculation dose led to a gradual decrease in pH and a progressive rise in titratable acidity of sour porridge inoculated with strain GXUN74722. When the inoculation dose exceeded 12.5%, both physicochemical indicators tended to stabilize, with the pH value maintained at 3.58 ± 0.02 and titratable acidity at 2.64 ± 0.07 °T. Such physicochemical shifts are intrinsically linked to the growth kinetics of LAB: insufficient inoculation dose slows LAB proliferation and restricts organic acid biosynthesis. By contrast, an excessively high inoculation dose leads to uneven substrate partitioning and limited proliferative niches, impairing cellular metabolic performance and acid-producing capacity of the strain (An et al., 2022). Therefore, balancing LAB growth kinetics and resource utilization efficiency, 12.5% was determined as the optimal inoculation dose for sour porridge fermentation with strain GXUN74722.

As a traditional fermented cereal product, sour porridge relies on cereal raw materials as the primary fermentation substrate, while readily available carbon sources including glucose, glycogen, and fructose serve as essential energy sources supporting microbial proliferation and central carbon metabolism. These carbon sources mediate microbial metabolic pathways and acid production rates, thereby ultimately influencing the types, contents and final flavor profiles of metabolites in the fermented products (Zhang et al., 2020; Zhang et al., 2024). Glucose, a readily catabolizable monosaccharide for LAB, imposes osmotic stress and suppresses bacterial propagation when supplied at excessive concentrations. According to previous studies, a 2% glucose stock solution was adopted as basal carbon supplement; accordingly, this work quantified how varying volume fraction of this 2% glucose stock solution in rice medium modulated key the physicochemical properties of fermented sour porridge. As shown in Figure 3C, incremental supplementation of the 2% glucose stock solution linearly lowered sample pH value while elevating titratable acidity. When the volume fraction reached 15%, the pH value dropped to 3.32 ± 0.02 and the titratable acidity increased to 4.35 ± 0.05 °T. The supplemented 2% glucose solution functions as a readily fermentable carbon substrate without causing excessive osmotic stress within the fermentation system, while simultaneously adjusting the moisture content of the matrix. Low moisture restricts full substrate solubilization and constrains LAB growth and metabolism, whereas elevated water availability stimulates LAB metabolic turnover and boosts organic acid yield (Li et al., 2023; Wang et al., 2023). Consequently, 15% volume fraction of the 2% glucose stock solution was determined as the optimal carbon supplementation level for sour porridge fermentation with strain GXUN74722.

Fermentation temperature dictates LAB proliferative capacity and metabolic activity throughout fermentation, critically modulating system physicochemical properties, fermentation efficiency and end-product quality (Valero-Cases et al., 2020). Different LAB possess specific optimal growth temperature range, within which robust vegetative growth is sustained (Zhang et al., 2019). Previous studies found that the optimal growth temperature of strain GXUN74722 was 30–37 °C, yielding cultures with pH value of 3.21–3.22 and titratable acidity of 4.2–4.28 °T. Building on these basal data, the present study further explored the effects of fermentation temperature on the physicochemical properties of sour porridge fermented by strain GXUN74722. As depicted in Figure 3D, rising fermentation temperature induced an initial decline followed by a rebound in sample pH, whereas titratable acidity exhibited a opposite trend with an early increase and subsequent reduction. The most pronounced acidification was detected at fermentation temperature of 34 °C and 37 °C, which exhibited the lowest pH and highest titratable acidity. Accordingly, the optimal temperature range for sour porridge fermentation with strain GXUN74722 was determined to be 34 °C-37 °C. Temperatures within this optimal range can potentiate the metabolic activity of strains, accelerate the biosynthesis of lactic acid and other acidic metabolite, and thereby refine the aromatic and gustatory profiles of fermented sour porridge. In contrast, supraoptimal thermal conditions inhibit the replication and metabolic activity of LAB, slowing substrate acidification (Meybodi et al., 2020). In agreement with our preliminary findings that identified 37 °C as the optimal growth temperature for strain GXUN74722, 37 °C was fixed as the fermentation temperature for subsequent sour porridge fermentation trials.

In aggregate, the optimal fermentation parameters for sour porridge fermentation with strain GXUN74722 were finalized as follows: fermentation time of 72 h, inoculation dose of 12.5%, volume fraction of 15% for the 2% glucose stock solution, and fermentation temperature of 37 °C.

### 3.4 Physicochemical properties of naturally fermented and inoculated fermented sour porridge

Naturally fermented sour porridge, a traditional fermented food in Guangxi, China, is currently mainly manufactured through small-scale household fermentation, resulting in inconsistent product quality. To tackle the drawbacks inherent to traditional fermented sour porridge and improve the quality and nutritional profiles of sour porridge products, this study investigated the differences in physicochemical properties and nutritional components between naturally fermented and GXUN74722-inoculated sour porridge.

**Figure 4:**
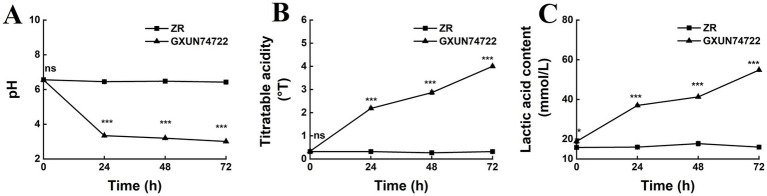
Changes in physicochemical properties of naturally fermented and GXUN74722-inoculated sour porridge. **(A)** pH; **(B)** Titratable acidity; **(C)** Lactic acid content. * means *p* < 0.05, ** means *p* < 0.01, *** means *p* < 0.001.

pH and titratable acidity serve as key indicators for assessing microbial acidification capacity, and their dynamic variations can comprehensively reflect the growth and metabolic profiles of LAB (Zhou et al., 2019). In the present study, sour porridge was inoculated with *L. paracasei* subsp. *paracasei* GXUN74722 and continuously fermented for 72 h under the optimized fermentation conditions determined in our previous experiments. As illustrated in Figures 4A,B, the pH and titratable acidity of naturally fermented sour porridge remained relatively stable throughout fermentation with no significant differences observed. In comparison, GXUN74722-inoculated sour porridge exhibited a pronounced decrease in pH and a remarkable elevation in titratable acidity (*p* < 0.001). After 72 h of fermentation, the pH value of GXUN74722-inoculated sour porridge decreased from 6.55 ± 0.02 to 3.01 ± 0.01, while titratable acidity increased from 0.33 ± 0.03 °T to 4.00 ± 0.50 °T. By contrast, naturally fermented sour porridge maintained a steady pH of approximately 6.56, and a constant titratable acidity of 0.32 °T, with negligible fluctuations during the entire fermentation cycles. Rapid variations in pH and titratable acidity were observed in the early fermentation stage (within the first 24 h) of GXUN74722-inoculated sour porridge. This phenomenon was primarily attributed to the vigorous metabolic activity of the inoculated LAB, which efficiently utilized carbohydrates in the fermentation substrate and rapidly produced organic acids. From 24 to 72 h of fermentation, the acid production rate gradually declined and eventually stabilized at 72 h. This trend was likely caused by nutrient depletion and accumulation of acidic metabolites in the fermentation system, which inhibited the proliferation and metabolic activity of LAB. Overall, the inoculated fermented sour porridge exhibited a substantial shift in pH (6.55 to 3.01) and titratable acidity (0.33 °T to 4.00 °T) over the fermentation process. These evident physicochemical alterations indicated that GXUN74722 inoculation significantly altered the acidification characteristics of sour porridge during fermentation.

Lactic acid is a characteristic metabolite produced by LAB during growth and metabolic processes. It not only mirrors the acid-producing capacity of LAB throughout fermentation but also serves as an indicator for judging the growth cycle of strains (Yang et al., 2025). As presented in Figure 4C, in the early fermentation stage (0–24 h), the lactic acid content of inoculated fermented sour porridge with strain GXUN74722 increased sharply with the extension of fermentation time, rising from 18.57 ± 1.25 mmol/L to 29.98 ± 0.56 mmol/L. During the mid-to-late fermentation phase, lactic acid accumulation continued to increase slowly and finally reached a plateau at 72 h, with a final concentration of 41.78 ± 0.31 mmol/L. In comparison, the lactic acid content of naturally fermented sour porridge remained stable throughout the whole fermentation process, only fluctuating in the range of 15.79–15.97 mmol/L. The significant difference in lactic acid accumulation patterns between the two fermentation systems could be explained by the abundant carbohydrate substrates in raw materials at the initial fermentation stage, which supported the rapid biosynthesis of lactic acid. The gradual consumption of available nutrients subsequently slowed down the acid synthesis rate of strains (Zhou et al., 2017). Collectively, these physicochemical results indicated that inoculation with GXUN74722 could rapidly activate LAB fermentation, significantly promote lactic acid accumulation, and a sharp decline in pH value of sour porridge. Based on the changes in physicochemical properties observed in this study, strain GXUN74722 inoculation has the potential to optimize the fermentation process of sour porridge and improve the physicochemical quality of fermented products.

### 3.5 Metabolic profiling

#### 3.5.1 Multivariate statistical analysis

**Figure 5:**
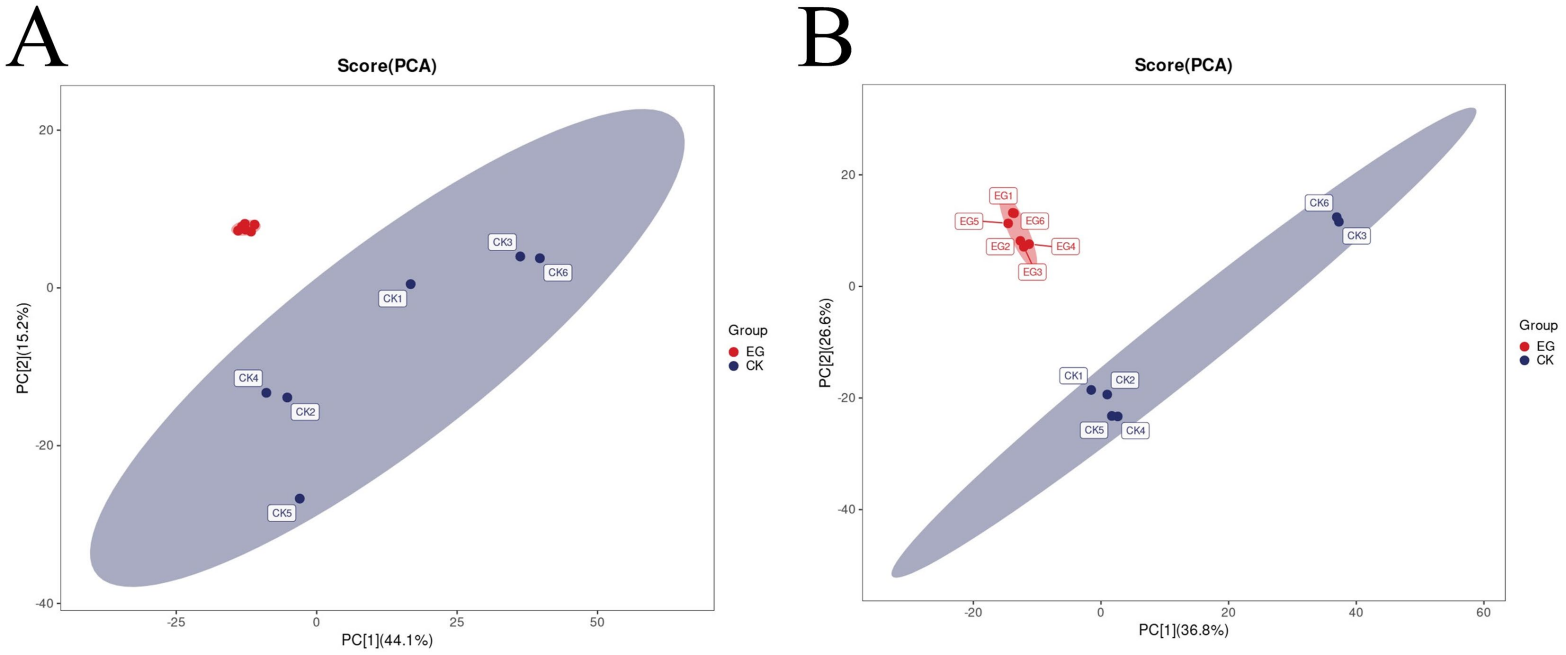
PCA score plots in positive ion and negative ion modes. **(A)** Positive ion modes; **(B)** Negative ion modes.

**Figure 6:**
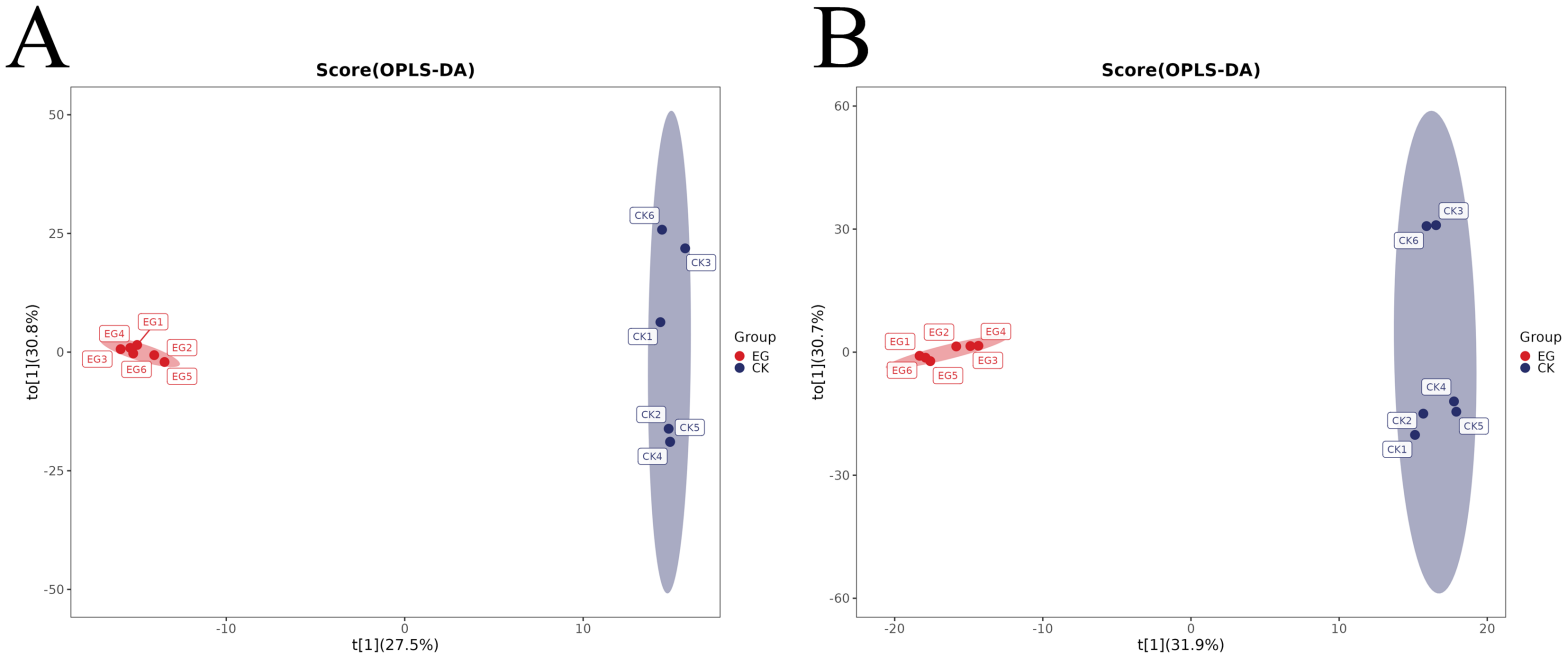
OPLS-DA score plots in positive ion and negative ion modes. **(A)** Positive ion modes; **(B)** Negative ion modes.

**Figure 7:**
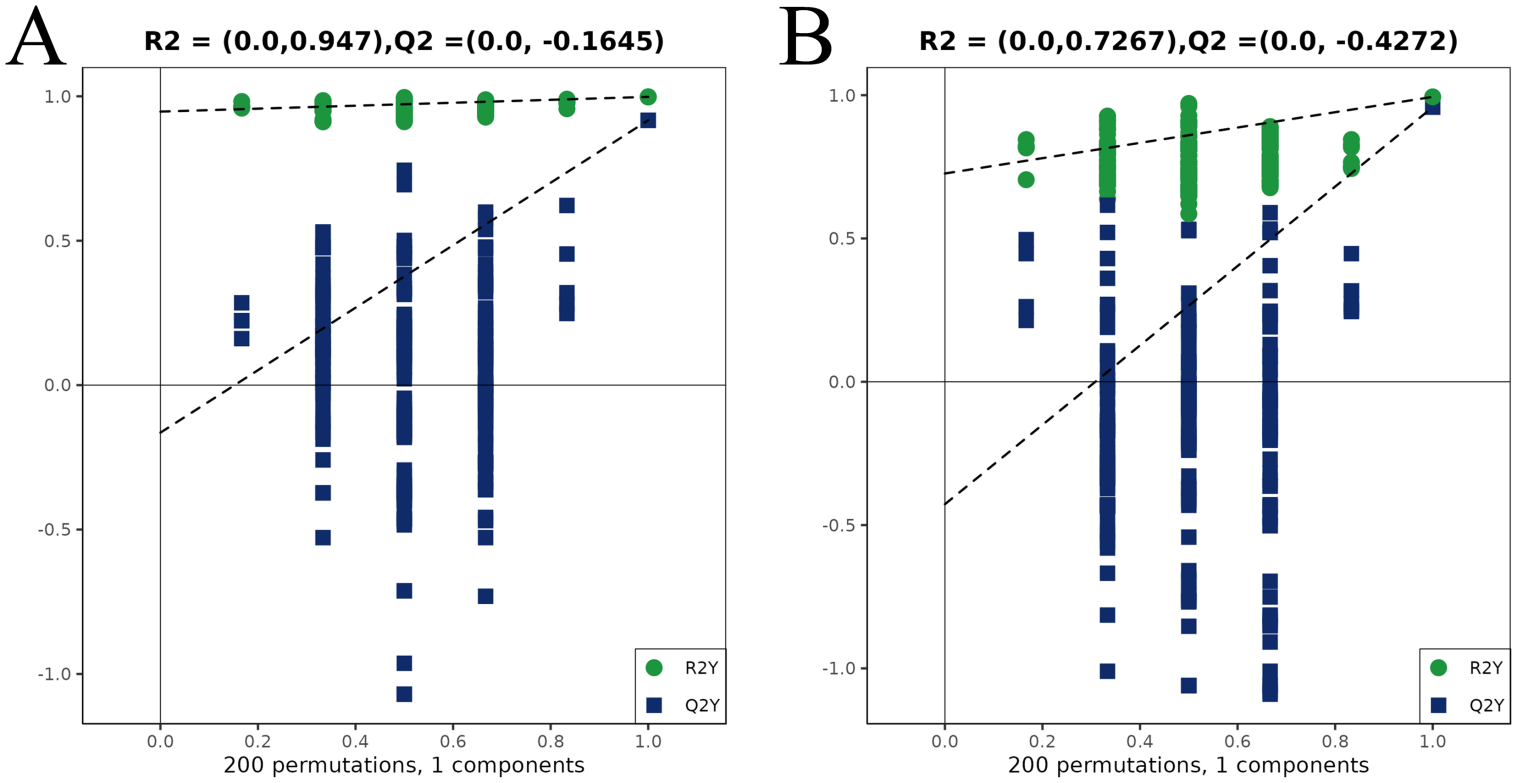
OPLS-DA permutation test plots in positive ion and negative ion modes. **(A)** Positive ion modes; **(B)** Negative ion modes.

Principal component analysis (PCA) score plots revealed that the model interpretation rates (R^2^X) of the CK groups and EG groups were 0.693 and 0.723, respectively, indicating a clear separation between samples based on both positive and negative ion mode datasets (Figure 5). Supervised orthogonal partial least squares discriminant analysis (OPLS-DA) was further performed to characterize the intergroup metabolic differences between CK and EG samples (Figure 6). The OPLS-DA model generated high fitness and predictive parameters, with R^2^Y = 0.998 and Q^2^ = 0.917 for the positive ion mode, and R^2^Y = 0.994 and Q^2^ = 0.958 for the negative ion mode. The values of R^2^Y and Q^2^ were both close to 1, indicating the high interpretation rate, strong prediction ability, favorable feasibility, and excellent fitting ability of the established model. Permutation tests were subsequently conducted to verify the reliability and avoid overfitting of the OPLS-DA model. In this model, R^2^Y and R^2^X represented the explanatory rates of the Y and X matrices, whereas Q^2^ reflected the model’s predictive performance. The intercept values of Q^2^ were calculated as −0.1645 for positive ion mode and −0.4272 for negative ion mode, both of which were below 0 (Figure 7). These results verified that the constructed OPLS-DA model exhibited no overfitting phenomenon and possessed excellent stability and robustness, which guaranteed the accuracy and credibility of subsequent differential metabolomic analysis between the CK and EG groups.

#### 3.5.2 Identification and characterization of metabolites in naturally fermented and inoculated fermented sour porridge

**Figure 8:**
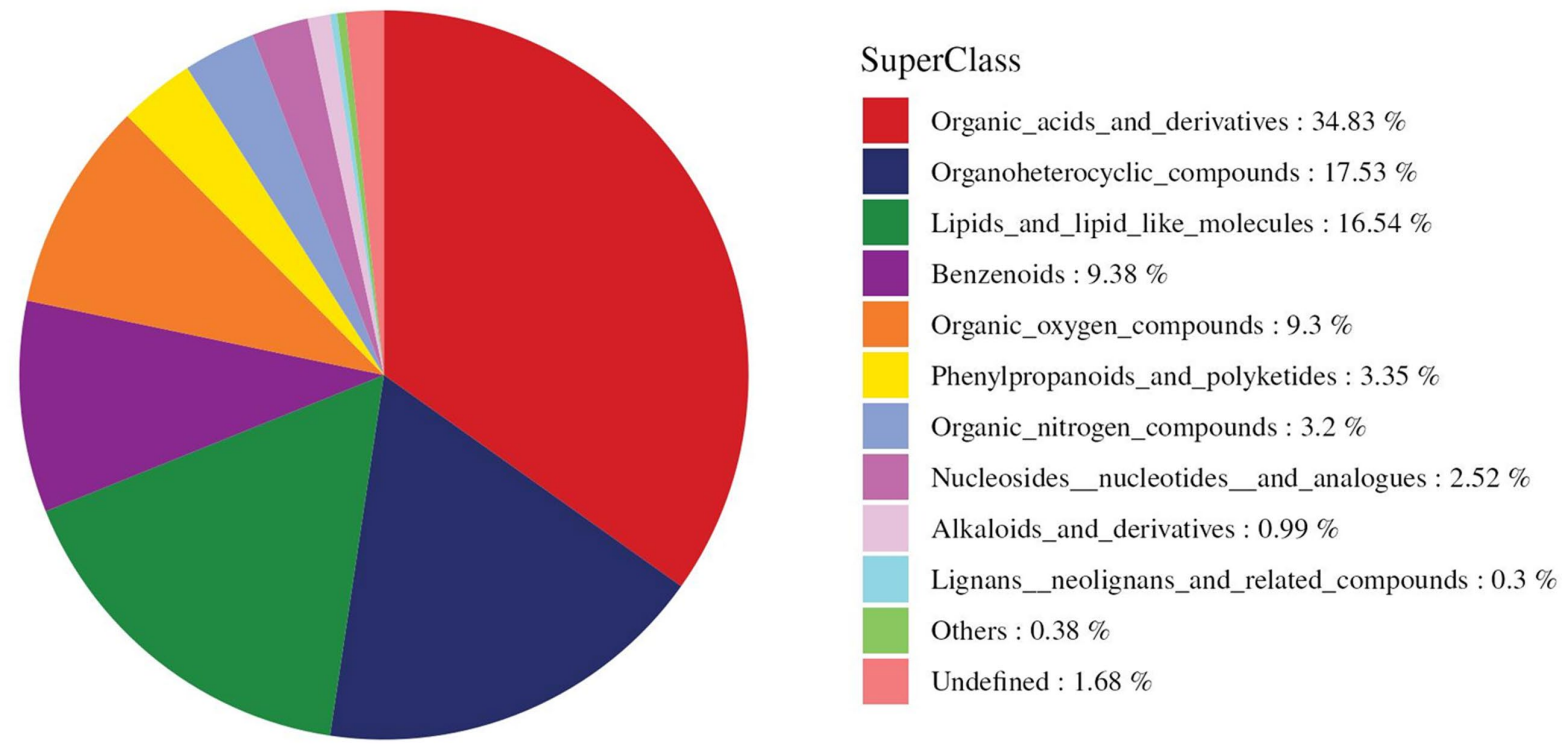
Proportion of identified metabolites across different chemical superclasses.

In this study, we systematically sorted and statistically analyzed the chemical classification of all identified metabolites and summarized the distribution characteristics of various metabolite categories. Metabolite classification visualization was performed at the Super Class level (Figure 8). The results showed that organic acids and derivatives dominated the identified metabolites, accounting for 34.83% of the total metabolites. The next predominant categories were organoheterocyclic compounds (17.53%), lipids and lipid-like molecules (16.54%), benzenoids (9.38%), organic oxygen compounds (9.3%), phenylpropanoids and polyketides (3.35%), organic nitrogen compounds (3.2%), nucleotides, nucleotides and analogues (2.52%), alkaloids and derivatives (0.99%), as well as lignans, neolignans and related compounds (0.3%). The remaining metabolites (2.06%) were categorized as other compounds.

Classification and quantitative analysis of the annotated metabolites identified a total of 1,312 metabolites across all samples, including 802 metabolites detected in positive ion mode and 510 in negative ion mode. Quantitative statistics showed that organic acids and derivatives (457 species), organoheterocyclic compounds (230 species), and lipids and lipid-like molecules (217 species) were the three most abundant metabolite classes. Organic acids and derivatives are closely correlated with the flavor characteristics and shelf-life properties of fermented foods. Their accumulation of these metabolites triggers a rapid decline in pH, which not only endows the unique flavor profile of sour porridge but also suppresses the proliferation of spoilage and pathogenic microorganisms. Most organoheterocyclic compounds possess complex molecular structures and can interact with diverse receptors and enzymes in biological systems. Typical representatives of this class, such as indoles and furans, have been recognized as key flavor-active substances contributing to the unique sensory properties of sour porridge (Guo et al., 2021). Lipids metabolites are also abundant in fermented products and profoundly modulate the taste and nutritional value of sour porridge. Specifically, unsaturated fatty acids (e.g., linoleic acid), alcohols, and ketones participate in the formation of sour taste characteristics (Chu et al., 2023) and optimize the nutritional composition of fermented products. Additionally, other metabolite categories, including organic nitrogen and organic oxygen compounds, are indispensable for numerous specific physiological and metabolic processes. Moreover, organosulfur compounds, nucleosides, nucleotides, and their analogues play critical roles in cellular energy metabolism, the structural assembly of biological macromolecules, and the biosynthesis of bioactive precursors.

#### 3.5.3 Cluster analysis of differential metabolites between naturally fermented and inoculated fermented sour porridge

**Figure 9:**
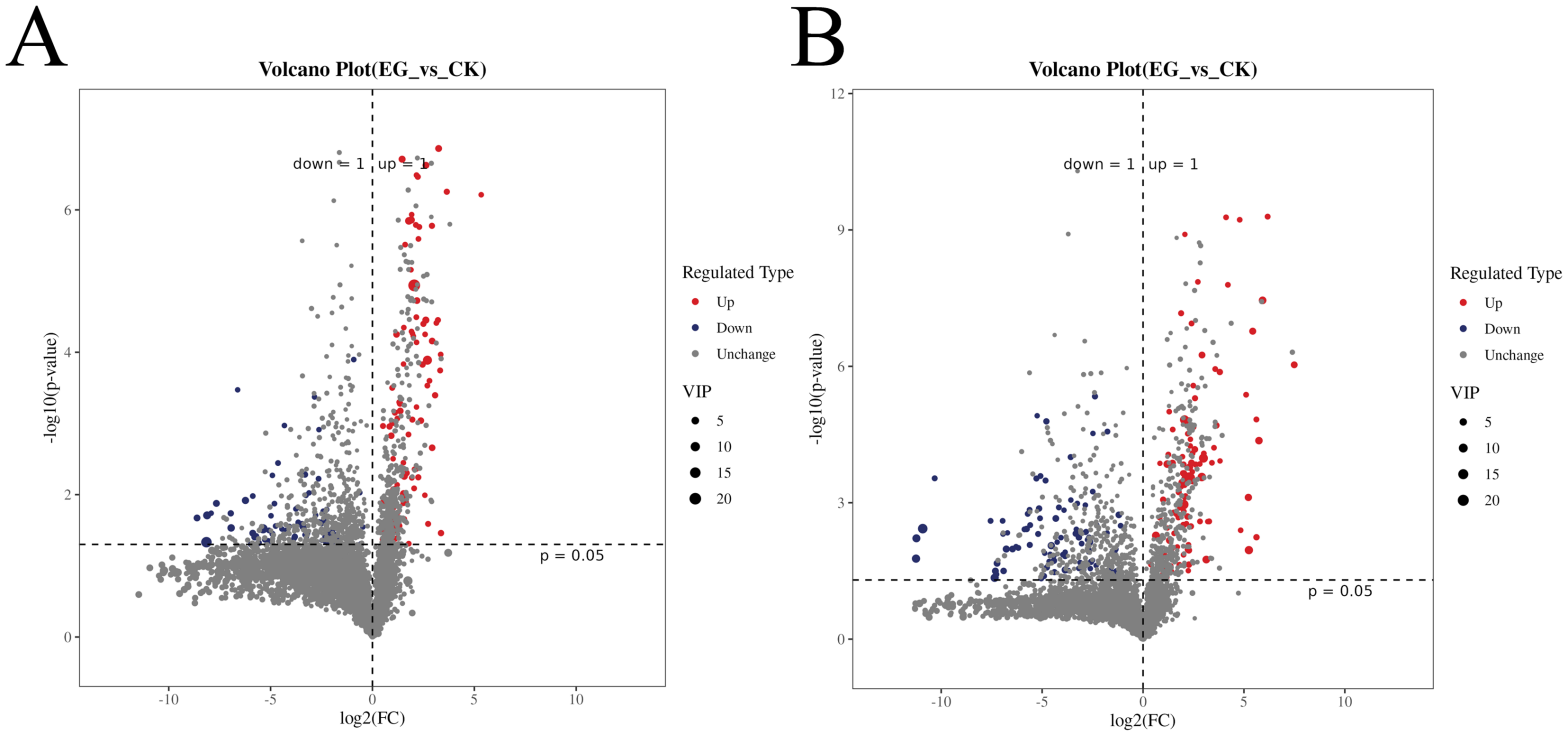
Volcano plots illustrating differentially expressed metabolites in positive and negative ion modes. **(A)** Positive ion modes; **(B)** Negative ion modes.

In the present study, metabolites satisfying the thresholds of VIP > 1 and *p* < 0.05 were identified as significantly differential metabolites. Furthermore, metabolites with FC > 1.5 were defined as up-regulated, while those with FC < 0.67 were defined as down-regulated. Based on the tandem mass spectrometry database annotation, a total of 83 differential metabolites were screened between naturally fermented sour porridge (CK group) and GXUN74722-inoculated sour porridge (EG group). Among these differential metabolites, 46 were identified in positive ion mode, including 30 up-regulated and 16 down-regulated metabolites (Figure 9A), and 37 were detected in negative ion mode, consisting of 19 up-regulated and 18 down-regulated metabolites (Figure 9B).

**Figure 10:**
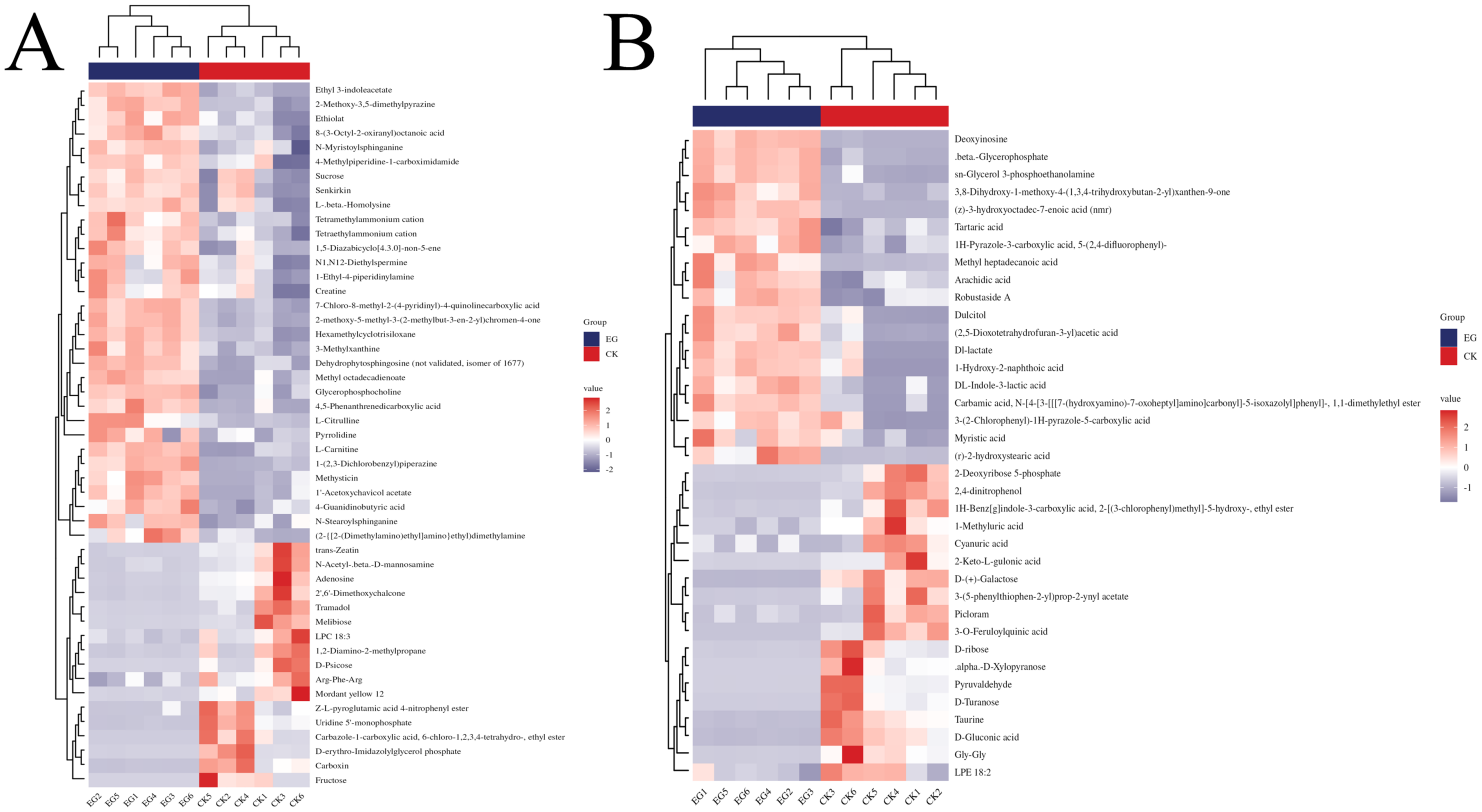
Hierarchical clustering heatmap of differentially expressed metabolites under positive and negative ion modes. **(A)** Positive ion modes; **(B)** Negative ion modes.

Cluster analysis was further performed on the 83 differential metabolites. The significantly changed metabolites between the CK and EG groups were primarily categorized into four classes: amino acids, peptides, and their analogues; carbohydrates and carbohydrate conjugates; lipids and lipid-like molecules; as well as benzene and substituted derivatives (Figure 10 and Table 1).

**Table 1:** VIP values of identified differential metabolites.

Form	Metabolite	RT	m/z	VIP	Fold change	Regulate
Amino acids, peptides and analogues	4-guanidinobutyric acid	386.410	128.082	1.313	2.484	↑
	L-citrulline	532.734	176.103	2.790	7.621	↑
	Arg-Phe-Arg	322.679	478.293	1.339	0.649	↓
	L-β-homolysine	737.068	144.102	1.487	1.843	↑
	creatine	483.785	132.077	1.244	1.936	↑
	Z-L-pyroglutamic acid 4-nitrophenyl ester	449.568	407.076	1.035	0.080	↓
	L-carnitine	490.984	162.112	4.104	3.739	↑
	Gly-Gly	450.960	131.045	1.054	0.040	↓
Carbohydrates and carbohydrate conjugates	sucrose	501.892	381.079	5.310	2.187	↑
	N-acetyl-β-D-mannosamine	268.612	126.055	1.122	0.101	↓
	melibiose	527.615	365.105	2.585	0.029	↓
	fructose	438.559	203.053	14.717	0.004	↓
	D-psicose	526.424	127.039	1.193	0.016	↓
	D-(+)-galactose	442.032	215.032	2.569	0.036	↓
	tartaric acid	46.978	149.008	1.649	2.382	↑
	dulcitol	439.257	181.071	4.923	4.932	↑
	D-gluconic acid	502.408	195.050	1.718	0.030	↓
	robustaside A	246.335	307.084	2.165	3.410	↑
	2-deoxyribose-5-phosphate	201.711	213.017	2.823	0.047	↓
	D-ribose	443.261	149.044	1.418	0.041	↓
	α-D-xylopyranose	441.913	113.023	3.722	0.006	↓
	2-keto-L-gulonic acid	223.549	175.024	2.117	0.033	↓
	D-turanose	442.51	161.044	3.609	0.007	↓
Lipids and lipid-like molecules	glycerophosphocholine	521.107	258.110	20.865	4.140	↑
	methyl octadecadienoate	136.276	263.237	1.864	5.661	↑
	8-(3-octyl-2-oxiranyl) octanoic acid	46.503	245.226	1.597	1.959	↑
	N-stearoylsphinganine	43.398	568.566	1.439	3.405	↑
	N-myristoylsphinganine	41.771	512.504	2.628	1.895	↑
	LPC 18:3	317.203	518.322	1.010	0.722	-
	β-glycerophosphate	519.432	171.005	1.408	6.575	↑
	sn-glycerol-3-phosphoethanolamine	524.903	214.048	2.082	5.961	↑
	methyl heptadecanoic acid	79.081	283.264	5.826	2.300	↑
	(r)-2-hydroxystearic acid	56.004	299.260	4.741	37.251	↑
	arachidic acid	50.292	311.295	1.8211	2.878	↑
	myristic acid	52.361	227.201	4.805	2.490	↑
	LPE 18:2	321.194	476.278	1.460	0.662	↓
Benzene and substituted derivatives	1-(3,4-dichlorobenzyl) piperazine	288.924	245.063	1.903	12.548	↑
	1′-acetoxychavicol acetate	375.842	257.074	1.750	9.277	↑
	4,5-phenanthrenedicarboxylic acid	441.718	205.068	9.031	6.492	↑
	1,4-oxathiine amide	440.132	218.063	1.453	0.040	↓
	trans-tramadol (or endogenous 3-methoxycyclohexylamine derivatives)	738.192	246.181	3.155	0.017	↓
	3,5-dinitrophenol (or 2,6-dinitrophenol)	262.838	183.006	1.296	0.060	↓

Fold changes represent significant increase or decrease in metabolite abundance. Compared with naturally fermented sour porridge, ↑ indicates up-regulation and ↓ indicates the down-regulation of differential metabolites.

Amino acids, peptides and their analogues are typical degradation products of proteins, which serve as pivotal metabolites in biological systems and are essential for maintaining fundamental cellular physiological activities. A total of eight differential metabolites were annotated in this category. Specifically, 4-guanidinobutyric acid, L-citrulline, L-*β*-homolysine, creatine, and L-carnitine were significantly up-regulated, whereas Arg-Phe-Arg and Gly-Gly were significantly down-regulated. Previous studies have confirmed that variations in these metabolites are closely related shelf-life extension, cholesterol regulation, gastrointestinal health maintenance, and flavor optimization of fermented products (Hwang and Jeong, 2012; Wu et al., 2007). Combined with the present results, it is speculated that proteins and amino acids in the sour porridge matrix act as crucial nitrogen source, supporting the growth and metabolic activity of strain GXUN74722 during fermentation. Strain-mediated metabolic transformation of these substrates not only sustains bacterial proliferation but also facilitates the biosynthesis of diverse bioactive components, thereby altering the metabolic characteristics of fermented sour porridge.

Carbohydrates and carbohydrate conjugates serve as key metabolic substrates for LAB during sour porridge fermentation. They not only modulate the growth and proliferation of LAB but also shape the characteristic flavor profiles of sour porridge. Among the 15 differential metabolites belonging to this category, sucrose, tartaric acid, dulcitol and robustaside A were significantly up-regulated, whereas melibiose, fructose, D-(+)-galactose and D-turanos were notably down-regulated. Such metabolic variations partially explain the elevated lactic acid accumulation in GXUN74722-inoculated sour porridge, thereby effectively guaranteeing the overall quality stability of sour porridge (Dong et al., 2022; Cui and Qu, 2021).

Lipids and lipid-like molecules constitute a class of lipid substances and their derivatives generated through enzymatic reactions between fatty acids and alcohols. These metabolites provide energy for biological metabolism, maintain cell structural integrity, and modulate the environmental adaptability of microorganisms, further influencing the nutritional component biosynthesis of sour porridge. Thirteen differential metabolites were annotated in the lipid category, among which 11 metabolites including glycerophosphocholine, sn-glycerol-3-phosphoethanolamine, arachidic acid and myristic acid were significantly up-regulated, while LPE 18:2 was significantly down-regulated. Such alterations in lipid metabolism modulate a variety of cellular functions and microbial environmental adaptability, ensuring strain survival throughout the fermentation process and further shaping the nutritional profile of in sour porridge (Han et al., 2024; Xu et al., 2017; Chamry et al., 2022). Collectively, these findings indicate that GXUN74722-inoculated sour porridge may possess superior nutritional properties relative to naturally fermented sour porridge.

Benzene and substituted derivatives are categorized into ether, acetoacetic acid, and carboxylic acid derivatives. Six differential metabolites were screened in this subclass, including three significantly up-regulated metabolites, with 1′-acetoxychavicol acetate as the typical representative. Previous studies have demonstrated that 1′-acetoxychavicol acetate possesses potential antiseptic properties for food application (Zhang et al., 2010). Collectively, these metabolite variations indicate that GXUN74722-inoculated sour porridge may help preserve product quality and nutritional composition as well as prolong shelf life, which is conductive to improving the edible quality of sour porridge.

Flavor is a crucial index for evaluating food palatability. During sour porridge fermentation, flavor-active compounds are primarily produced from primary metabolites via core metabolic pathways, including carbohydrate metabolism, lipolysis, and proteolysis (Yang et al., 2024). As illustrated in Figure 10 and Table 2, a total of 19 acids, 5 esters, 5 heterocyclic compounds, 3 ketones, 1 ether, 1 phenol, and 1 terpenoid were identified in all samples. Several characteristic flavor substances, including DL-lactate, robustaside A, 1′-acetoxychavicol acetate, ethyl-3-indoleacetate, and 2-methoxy-3,5-dimethylpyrazine were significantly up-regulated in the GXUN74722-inoculated sour porridge. The differential accumulation of these flavor-active metabolites contributes substantially to the formation and optimization of the unique flavor profile of fermented sour porridge.

**Table 2:** Variations in volatile flavor and aromatic compounds.

Form	Metabolite	RT	m/z	VIP	Fold change	Regulate
Acid	7-chloro-8-methyl-2-(4-pyridinyl)-4-quinolinecarboxylic acid	40.048	299.062	1.332	4.479	↑
	4,5-phenanthrenedicarboxylic acid	441.718	205.068	9.031	6.492	↑
	8-(3-octyl-2-oxiranyl) octanoic acid	46.503	245.226	1.597	1.959	↑
	4-guanidinobutyric acid	386.410	128.082	1.313	2.484	↑
	(z)-3-hydroxyoctadec-7-enoic acid (nmr)	129.303	297.244	5.886	60.681	↑
	(2,5-dioxotetrahydrofuran-3-yl) acetic acid	564.726	175.024	1.147	5.065	↑
	DL-indole-3-lactic acid	272.260	204.066	1.668	5.476	↑
	tartaric acid	46.978	149.008	1.649	2.382	↑
	DL-lactate	349.131	89.023	24.443	4.873	↑
	D-gluconic acid	502.408	195.050	1.718	0.030	↓
	(r)-2-hydroxystearic acid	56.004	299.260	4.741	37.250	↑
	arachidic acid	50.292	311.295	1.821	2.878	↑
	3-O-feruloylquinic acid	444.006	367.105	12.634	0.001	↓
	myristic acid	52.361	227.201	4.805	2.490	↑
	1-methyluric acid	316.351	363.074	1.830	0.157	↓
	3-(2-chlorophenyl)-1H-pyrazole-5-carboxylic acid	38.787	221.012	1.199	2.649	↑
	2-keto-L-gulonic acid	223.549	175.024	2.117	0.033	↓
	cyanuric acid	443.159	128.009	1.916	0.410	↓
	methyl heptadecanoic acid	79.081	283.264	5.826	2.300	↑
Ether	trans-tramadol (or endogenous 3-methoxycyclohexylamine derivatives)	738.192	246.181	3.155	0.017	↓
Phenol	3,5-dinitrophenol (or 2,6-dinitrophenol)	262.838	183.001	1.296	0.060	↓
Terpenoids	robustaside A	246.335	307.084	2.165	3.410	↑
Ketone	2-methoxy-5-methyl-3-(2-methylbut-3-en-2-yl) chromen-4-one	40.718	297.082	3.199	6.173	↑
	2′,6′-dimethoxychalcone	303.636	269.108	1.105	0.145	↓
	3,8-dihydroxy-1-methoxy-4-(1,3,4-trihydroxybutan-2-yl) xanthen-9-one	42.918	313.078	2.864	12.412	↑
Ester	1′-acetoxychavicol acetate	375.842	257.074	1.750	9.277	↑
	methyl octadecadienoate	136.276	263.237	1.864	5.661	↑
	D-erythro-imidazolylglycerol phosphate	297.532	111.056	2.262	0.071	↓
	Z-L-pyroglutamic acid 4-nitrophenyl ester	449.568	407.076	1.035	0.080	↓
	carbamic acid, N-[4-[3-[[[7-(hydroxyamino)-7-oxoheptyl]amino]carbonyl]-5-isoxazolyl]phenyl]-, 1,1-dimethylethyl ester	438.278	371.137	3.477	5.576	↑
Heterocyclic	ethyl 3-indoleacetate	523.846	226.084	3.235	9.502	↑
	2-methoxy-3,5-dimethylpyrazine	316.287	111.092	1.042	3.712	↑
	carbazole-1-carboxylic acid, 6-chloro-1,2,3,4-tetrahydro-, ethyl ester	444.046	204.056	5.798	0.004	↓
	1H-benz[g]indole-3-carboxylic acid, 2-[(3-chlorophenyl)methyl]-5-hydroxy-, ethyl ester	286.856	378.085	1.016	0.041	↓
	3-(5-phenylthiophen-2-yl) prop-2-ynyl acetate	245.261	255.047	1.105	0.034	↓

Fold changes represent significant increase or decrease in metabolite abundance. ↑ indicates the up-regulation of differential metabolites compared with naturally fermented sour porridge. ↓ indicates the down-regulation of differential metabolites.

#### 3.5.4 KEGG functional annotation and enrichment analysis of differential metabolites in naturally fermented and inoculated fermented sour porridge

**Figure 11:**
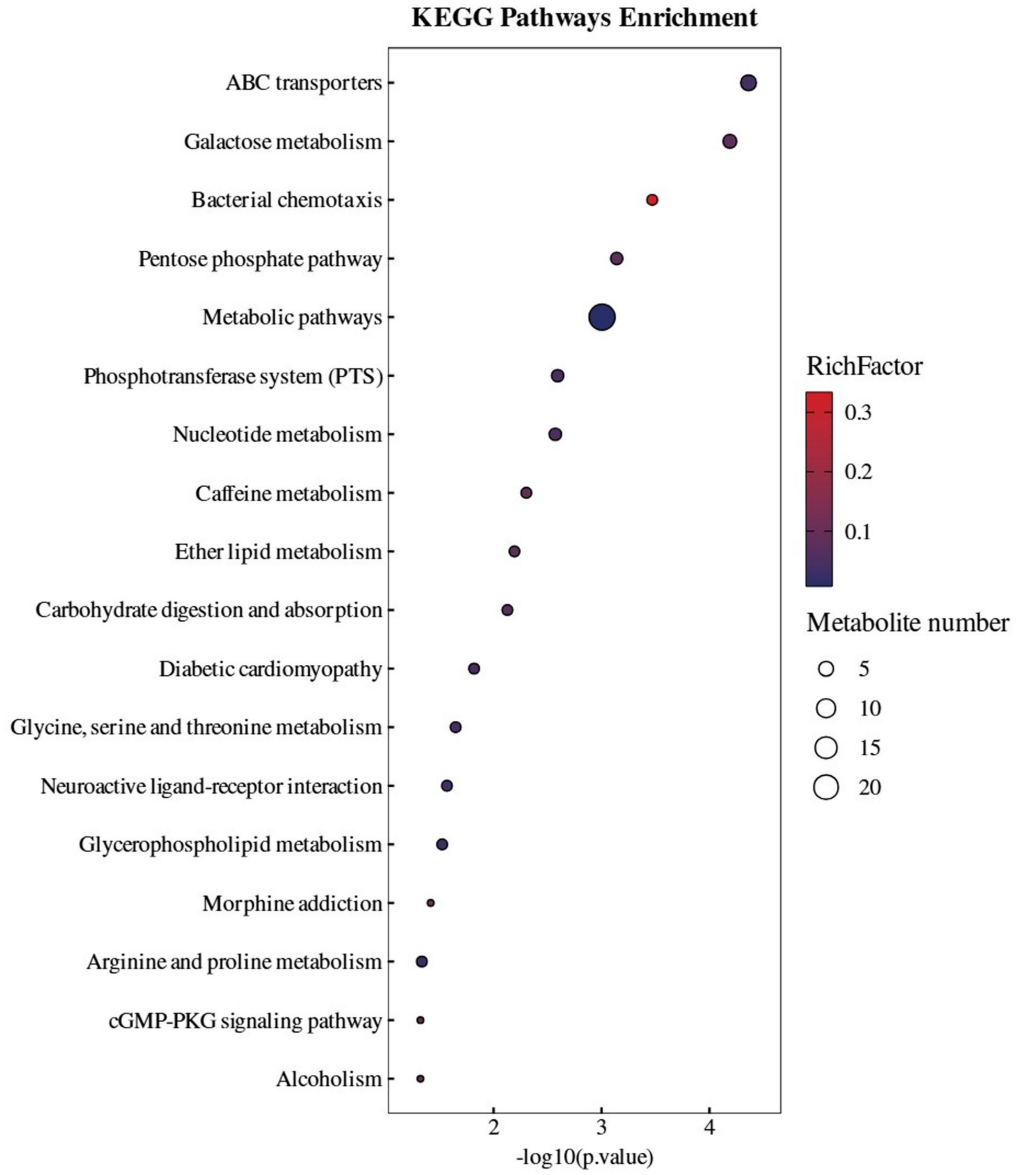
KEGG enrichment analysis of differential metabolites.

The KEGG database enables systematic annotation metabolite-related pathways and facilitates the identification of key regulatory nodes in microbial metabolism. In this study, KEGG functional pathway enrichment analysis was performed on significantly differential metabolites (VIP > 1, *p* < 0.05) screened from the CK and EG groups. Annotated metabolic pathways were categorized into four major classes, consisting of 82 metabolism-related pathways, 9 organismal system pathways, 15 genetic information processing pathways, and 2 cellular processes pathways. These analytical results revealed that differentially enriched KEGG pathways were mainly clustered into material and energy metabolism as well as genetic information processing categories. The enrichment profile implied that shifts in metabolite profiles were correlated with active substance and energy metabolic processes, whereas pathways related to genetic information metabolism exhibited relatively minor variation. Meanwhile, obvious distinctions in enriched metabolic pathways were found between naturally fermented and GXUN74722-inoculated fermentation groups, which was associated with divergent metabolic phenotypes and environmental adaptation characteristics of microbial communities. Based on the enrichment threshold of *p* < 0.05, the top 20 significantly enriched differential metabolic pathways were screened, among which ABC transporters, galactose metabolism, pentose phosphate pathway, carbohydrate digestion and absorption, and glycerophospholipid metabolism exhibited the most prominent enrichment (Figure 11). These findings showed that differential metabolites were significantly enriched in the above core metabolic pathways. Such enrichment patterns are correlated with the fermentation process of sour porridge, and targeted modulation of these pathways may be linked to changes in fermentation performance. These observations provide tentative clues for exploring metabolic features underlying LAB-mediated sour porridge fermentation.

**Figure 12:**
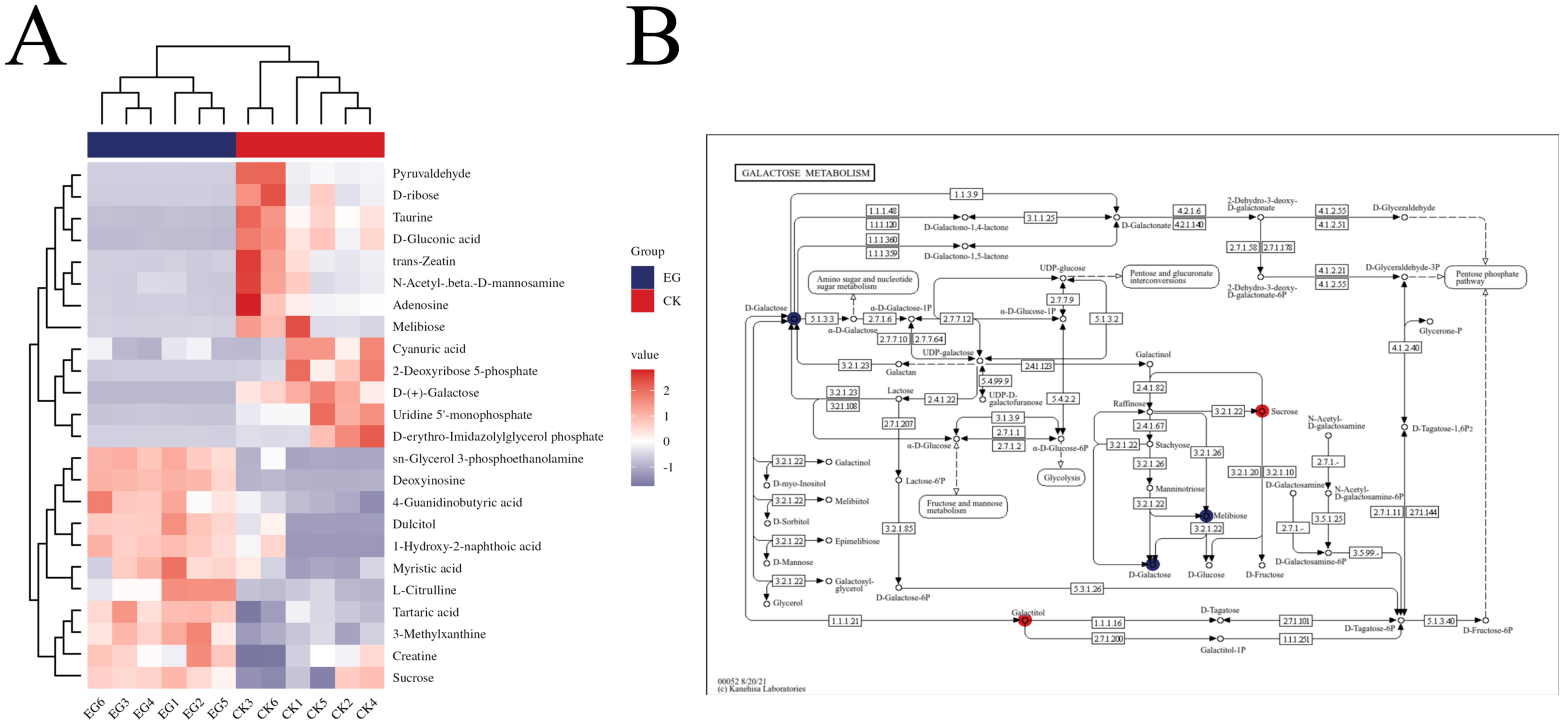
Metabolic pathway analysis. **(A)** Cluster heatmap of differential metabolites involved in metabolic pathways; **(B)** Schematic diagram of the galactose metabolism pathway.

Previous studies have verified that ABC transporters mediate the transmembrane transport of multiple substrates, including sugars, amino acids, and proteins (Keresztény et al., 2024). As shown in Figure 12A, in the ABC transporter pathway, the relative contents of sucrose, D-ribose, adenosine, taurine, and melibiose decreased in the GXUN74722-inoculated group, while deoxyinosine levels increased. This metabolic variation indicated that LAB utilized carbohydrates as the primary energy sources for ATP synthesis during strain-driven fermentation. The catabolic intermediates were further converted into diverse nutritional components and flavor compounds, supporting the growth and metabolic activity of LAB (He C. L. et al., 2025). Moreover, three differential metabolites enriched in the pentose phosphate pathway, namely D-(+)-galactose, D-gluconic acid, and 2-deoxyribose 5-phosphate, exhibited active metabolic characteristics during fermentation. These metabolites are correlated with amino acids and fatty acids biosynthetic pathways according to previous research (Rashida and Laxman, 2021), providing tentative clues for understanding the divergent quality performance between inoculated and naturally fermented sour porridge groups. Carbohydrate metabolism serves as the core metabolic pathway of fermented sour porridge, wherein LAB predominantly generate lactic acid and metabolic energy via carbohydrate catabolism. The significant enrichment of the galactose metabolism pathway in this study was closely related to the consumption and transformation of galactose substrates during sour porridge fermentation (Iskandar et al., 2019). After GXUN74722-inoculated fermentation, the relative levels of sucrose and galactitol elevated, whereas melibiose and D-(+)-galactose decreased, which may be attributed to the biotransformation of raffinose. Notably, previous studies have documented multiple bioactivity potentials of raffinose and galactitol, including possible effects on intestinal microbiota composition and mineral uptake. The accumulation of these two metabolites in fermented sour porridge may correlate with altered functional-related traits of the final product (Kanwal et al., 2023; Liu et al., 2024). Glycerophospholipid metabolism is an essential lipid metabolic pathway containing subpathways for phosphatidylcholine and phosphatidylethanolamine biosynthesis. In this study, most differential metabolites annotated to glycerophospholipid metabolism, such as glycerophosphocholine and sn-glycerol-3-phosphoethanolamine, clustered within the phosphatidylethanolamine biosynthetic branch. Existing literature suggests these compounds are linked to a range of potential bioactivities (Zhang et al., 2022). Furthermore, existing literature indicates that glycerophospholipid metabolism correlates with lipid breakdown processes and may be linked to nutritional-related attributes of fermented foods. Lan et al. (2025) observed dynamic fluctuations of phospholipids ethanolamine and phosphatidylcholine during rice bran fermentation. Chen et al. (2022) employed lipidomics to analyze variations in flavor precursors of purple leaf tea under different processing treatments, and their findings implied glycerophospholipid metabolism is among the core lipid-related metabolism pathways associated with tea processing. Additionally, the level of 4-guanidine-butyric acid was significantly up-regulated in the relevant metabolic pathways, and this metabolite had reported its potential inhibitory effect against the pathogenic bacteria *Helicobacter pylori* (Hwang and Jeong, 2012), implying the elevated content of this metabolite could be correlated with altered antibacterial-related properties of sour porridge. Tartaric acid, as a natural food additive with preservative potential, can interact with food proteins in earlier studies (Wu et al., 2024), hence the enrichment of tartaric acid in fermented samples provides tentative clues relevant to the variation in product stability and shelf-life traits of sour porridge.

In summary, carbohydrates constitute the primary carbon and energy sources for LAB growth during sour porridge fermentation. As a key carbohydrate, galactose can be catabolized via the Leloir pathway and the tagatose-6-phosphate pathway, before entering the central glycolytic pathway (Wang Y. et al., 2021). As illustrated in Figure 12B, the accumulation of polysaccharide-related components in GXUN74722-inoculated sour porridge correlates with the metabolic transformation of sucrose, melibiose, D-galactose and galactitol. Existing literature identifies D-(+)-galactose as a pivotal intermediate and hub metabolite in galactose metabolism that connects core glucose metabolism to diverse downstream metabolic pathways. The observed variation of this metabolite alongside shifts in glucose metabolism and polysaccharide biosynthesis provides tentative clues linking galactose metabolic changes to differing profiles of flavor-associated metabolites and divergent overall quality traits between fermented sour porridge.

## 4 Conclusion

This work explored the metabolic profiles linked to product traits of sour porridge fermented with *Lactobacillus paracasei* subsp. *paracasei* GXUN74722. Fermentation condition optimization was performed based on physicochemical indicators, and the optimal process parameters for strain GXUN74722 were determined as 72 h of fermentation time, 12.5% inoculation dose, 37 °C of fermentation temperature, and 15% volume fraction supplementation of 2% glucose solution. Compared with naturally fermented sour porridge, the inoculated fermented sour porridge with strain GXUN74722 fermented for 72 h exhibited significantly altered physicochemical properties: with a pH of 3.01 ± 0.01, titratable acidity of 4.00 ± 0.5 °T, and lactic acid content of 41.78 ± 0.31 mmol/L. The above parameter variations suggest that strain inoculation correlates with changed fermentation cycle and modified physicochemical profiles of sour porridge. Untargeted metabolomics analysis revealed a rich and diverse array of metabolites in the sour porridge. Among these metabolites, organic acids and derivatives accounted for the largest proportion (34.83%), followed by organoheterocyclic compounds (17.53%), lipids and lipid-like molecules (16.54%), benzenoids (9.38%), organic oxygen compounds (9.3%), phenylpropanoids and polyketides (3.35%), organic nitrogen compounds (3.2%), nucleosides, nucleotides and analogues (2.52%), alkaloids and derivatives (0.99%), lignans, neolignans and related compounds (0.3%), with the remaining 2.06% belonging to other categories. A total of 83 significantly differentiated metabolites were screened via positive and negative ion modes, mainly including amino acids, peptides and analogues; carbohydrates and carbohydrate conjugates; lipids and lipid-like molecules; and benzene and substituted derivatives. Among the volatile flavor compounds, acids, esters and heterocyclics were the dominant flavor-related components in the inoculated fermented sour porridge. KEGG pathway enrichment analysis demonstrated that these differential metabolites were primarily enriched in key biosynthesis and metabolic pathways, including those related to amino acids, carbohydrates, organic acids, nucleotides, and glycerophosphoric acid. These metabolic pathways are correlated with divergent flavor and nutritional-related phenotypes of sour porridge. Overall, this study characterized distinct metabolic signatures of natural fermentation and GXUN74722-inoculated fermentation. The dataset can serve as preliminary technical references for developing pure-strain fermented sour porridge, and lay groundwork for subsequent investigation on industrial quality modulation. Additional sensory evaluation and in-depth functional verification experiments are needed in future work to confirm whether strain inoculation brings favorable changes in flavor and nutritional performance of finished products.

## Data Availability

The datasets used in the current study are available from the corresponding author on reasonable request. Full-length sequence of 16S rRNA gene of *Lacticaseibacillus paracasei* subsp. paracasei GXUN74722 has been deposited in the NCBI GenBank database under the accession number PX670456 (https://www.ncbi.nlm.nih.gov/nuccore/PX670456).

## Footnotes

1
https://www.ezbiocloud.net
